# Emergence of Neuronal Synchronisation in Coupled Areas

**DOI:** 10.3389/fncom.2021.663408

**Published:** 2021-04-22

**Authors:** Paulo R. Protachevicz, Matheus Hansen, Kelly C. Iarosz, Iberê L. Caldas, Antonio M. Batista, Jürgen Kurths

**Affiliations:** ^1^Applied Physics Department, Institute of Physics, University of São Paulo, São Paulo, Brazil; ^2^Computer Science Department, Institute of Science and Technology, Federal University of São Paulo - UNIFESP, São José dos Campos, Brazil; ^3^Faculdade de Telêmaco Borba, Telêmaco Borba, Brazil; ^4^Graduate Program in Chemical Engineering, Federal University of Technology Paraná, Ponta Grossa, Brazil; ^5^Department of Mathematics and Statistics, State University of Ponta Grossa, Ponta Grossa, Brazil; ^6^Department Complexity Science, Potsdam Institute for Climate Impact Research, Potsdam, Germany; ^7^Department of Physics, Humboldt University, Berlin, Germany; ^8^Centre for Analysis of Complex Systems, Sechenov First Moscow State Medical University, Moscow, Russia

**Keywords:** synchronisation, excitatory and inhibitory connections, exponential adaptive integrate-and-fire model, neuronal activities, coupled areas

## Abstract

One of the most fundamental questions in the field of neuroscience is the emergence of synchronous behaviour in the brain, such as phase, anti-phase, and shift-phase synchronisation. In this work, we investigate how the connectivity between brain areas can influence the phase angle and the neuronal synchronisation. To do this, we consider brain areas connected by means of excitatory and inhibitory synapses, in which the neuron dynamics is given by the adaptive exponential integrate-and-fire model. Our simulations suggest that excitatory and inhibitory connections from one area to another play a crucial role in the emergence of these types of synchronisation. Thus, in the case of unidirectional interaction, we observe that the phase angles of the neurons in the receiver area depend on the excitatory and inhibitory synapses which arrive from the sender area. Moreover, when the neurons in the sender area are synchronised, the phase angle variability of the receiver area can be reduced for some conductance values between the areas. For bidirectional interactions, we find that phase and anti-phase synchronisation can emerge due to excitatory and inhibitory connections. We also verify, for a strong inhibitory-to-excitatory interaction, the existence of silent neuronal activities, namely a large number of excitatory neurons that remain in silence for a long time.

## 1. Introduction

The study of synchronisation of neuronal activities is one of the greatest topics in neuroscience (Achuthan and Canavier, 2009; Fell and Axmacher, 2011; Protachevicz et al., 2020). Vysata et al. (2014) analysed synchronous behaviour between different areas through electroencephalogram (EEG) time series. The existence of phase, anti-phase, and shift-phase synchronisation between brain areas during different cognitive tasks have been reported in many works (Luo and Guan, 2018; Alagapan et al., 2019; Carlos et al., 2020). Due to this fact, the capability of neurons to synchronise in phase and anti-phase has been broadly investigated (Achuthan and Canavier, 2009; Liang et al., 2009; Belykh et al., 2010; Jalil et al., 2010, 2012; Batista et al., 2012; Wang et al., 2012; Ao et al., 2013; Lowet et al., 2016; Kim and Lim, 2020).

Phase synchronisation between brain regions was observed during memory processes (Klimesch et al., 2008; Fell and Axmacher, 2011; Polanía et al., 2012; Fell et al., 2013; Clouter et al., 2017; Daume et al., 2017; Staudigl et al., 2017; Bahramisharif et al., 2018; Gruber et al., 2018), perception (Jamal et al., 2015), attention (Sauseng et al., 2008; Kwon et al., 2015), and motor tasks (Serrien and Brown, 2002). It was also reported for subjects playing guitar (Lindenberger et al., 2009), meditating (Herbert et al., 2005; Josipovic et al., 2012), in conscious perception (Melloni et al., 2007), and during cognitive processes (Canolty et al., 2006). Phase and anti-phase were observed in the monkey visual cortex (Spaak et al., 2012). The organisation of anti-phase synchronisation can be related to delayed excitatory conductance between regions (Knoblauch et al., 2003; Li and Zhou, 2011; Petkoski et al., 2018, 2019). The results demonstrated by Fox et al. (2005) suggest that anticorrelated activities in the brain dynamics, as well as correlated activities, can arise naturally in the human brain. Some works have also reported observations of anticorrelated activities in the mammalian brain (Fox et al., 2009; Josipovic et al., 2012; Liang et al., 2012; Schwarz et al., 2013; Kodama et al., 2018).

Recently, the synchronisation in neuronal networks in presence of both excitatory and inhibitory synapses has been observed using neuronal models (Bera et al., 2019a; Pal et al., 2021) coupled through hypernetworks (Rakshit et al., 2018a; Bera et al., 2019b) and multiplex configurations (Rakshit et al., 2018b). In cortico-cortical communication, one cortical area can interact with other one by means of excitatory and inhibitory connectivities (Roland et al., 2014; Tamioka et al., 2015; Tovete et al., 2015; D'Souza et al., 2016). In this work, we investigate how the excitatory and inhibitory connectivities from one area to another influence the phase angle and neuronal synchronisation. We consider unidirectional (sender-receiver) and bidirectional interactions between two areas. For the unidirectional interaction and desynchronised neurons in the sender area, we show that the phase angle values and synchronous behaviour of the neurons in the receiver area depend not only on the neuronal dynamics of the sender area, but also on the type of connections between the areas. We find phase, anti-phase, and shift-phase synchronisation in the receiver area when the neurons are synchronised in the sender one. With regard to the bidirectional interaction, we verify phase and anti-phase synchronous behaviour between the areas. The excitatory-to-excitatory (inhibitory-to-inhibitory) and excitatory-to-inhibitory (inhibitory-to-excitatory) connections can induce phase and anti-phase synchronisation between the areas, respectively. For a strong inhibitory-to-excitatory interaction between the areas, a large number of silent excitatory neurons are found in both areas.

The paper is organised as follows. In section 2, we introduce the neuronal network composed of adaptive exponential integrate-and-fire (AEIF) neurons and the diagnostic tools to characterise the synchronous behaviour. Sections 3 and 4 present our results and discussions about the effects of the unidirectional and bidirectional interactions between two areas, respectively, on the neuronal synchronisation and the phase angle. We draw our conclusions in section 5.

## 2. Methods

### 2.1. Network

**Figure 1:**
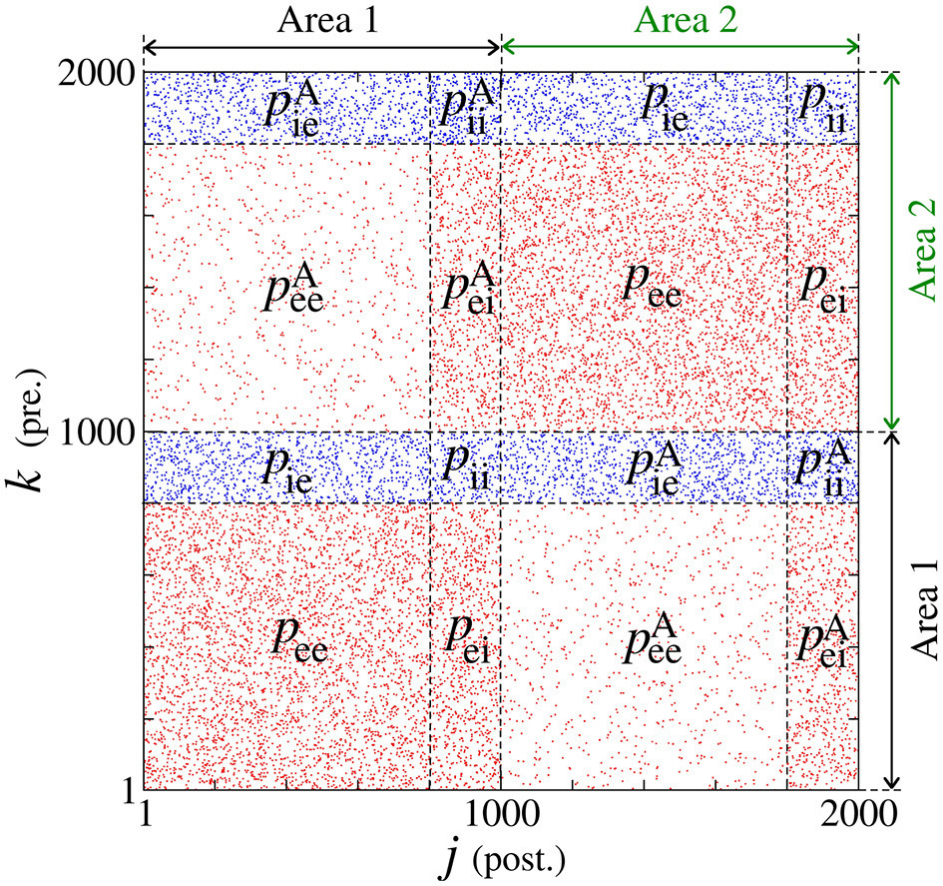
Connection matrix of two areas, where excitatory and inhibitory neurons are randomly connected. In each area, *p*_ee_ and *p*_ii_ are the probabilities of connection between excitatory and inhibitory neurons, respectively. The probability *p*_ei_ (*p*_ie_) corresponds to the connection from the excitatory (inhibitory) to inhibitory (excitatory) neurons. The superscript “A” is used for the connection probabilities between neurons in different areas.

We build a neuronal network composed of two areas, where each one has a thousand of adaptive exponential integrate-and-fire neurons (*N* = 1, 000) (Brette and Gerstner, 2005). Each area has a fraction of excitatory (*P*_exc_ = 0.8) and inhibitory (*P*_inh_ = 0.2) neurons (Noback et al., 2005; di Volo et al., 2019). In each area, the neurons are randomly coupled by means of excitatory and inhibitory connections. The connection is excitatory (inhibitory) when it occurs from an excitatory (inhibitory) neuron. Inside of each area, the probabilities of connections in the same neuronal populations (excitatory or inhibitory) are given by *p*_ee_ = 0.05 and *p*_ii_ = 0.2, while between different neuronal populations by *p*_ei_ = *p*_ie_ = 0.05 (di Volo et al., 2019). Between the areas, the probabilities are given by $p_{\text{ee}}^{\text{A}}=0.01$ (from excitatory to excitatory neurons), $p_{\text{ei}}^{\text{A}}=0.05$ (from excitatory to inhibitory neurons), $p_{\text{ii}}^{\text{A}}=0.10$ (from inhibitory to inhibitory neurons), and $p_{\text{ie}}^{\text{A}}=0.05$ (from inhibitory to excitatory neurons). Figure 1 shows how the probabilities are distributed in a connection matrix, where *k* and *j* correspond to the presynaptic and postsynaptic neurons, respectively. In Figure 1, *j, k* ∈ [1, 1000] correspond to the neurons in Area 1 and *j, k* ∈ [1001, 2000] to the neurons in Area 2. For the unidirectional configuration, the connections given by *k* = [1001, 2000] and *j* = [1, 1000] are not considered. For both unidirectional and bidirectional configuration, we consider only excitatory or inhibitory connections between the areas in each case.

With regard to the coupling intensities, each one is associated with a probability of connection and denoted by *g*_ee_, *g*_ii_, *g*_ei_, *g*_ie_, $g_{\text{ee}}^{\text{A}}$, $g_{\text{ei}}^{\text{A}}$, $g_{\text{ii}}^{\text{A}}$, and $g_{\text{ie}}^{\text{A}}$. For instance, *g*_ei_ and *g*_ie_ are related to the connections with the probabilities *p*_ei_ and *p*_ie_, respectively.

### 2.2. Neuronal Model

The cortex is mainly constituted by excitatory pyramidal neurons and inhibitory interneurons (Atencio and Schreiner, 2008). Excitatory neurons have a relatively lower firing rate than inhibitory ones (Wilson et al., 1994; Inawashiro et al., 1999; Baeg et al., 2001). In the mammalian cortex, excitatory neurons show regular spike (RS), while inhibitory neurons exhibit fast spike (FS) activities (Neske et al., 2015; Wang et al., 2016). In addition, while inhibitory neurons exhibit a negligible adaptation, excitatory neurons show an adaptation mechanism in their firings (Foehring et al., 1991; Mancilla et al., 1998; Hensch and Fagiolini, 2004; Destexhe, 2009; Masia et al., 2018; Borges et al., 2020). The adaptive exponential integrate-and-fire (AEIF) model is able to mimic these different firing patterns, including RS and FS (di Volo et al., 2019). In this work, the dynamics of each neuron *j* (*j* = 1, …, *N*) in the network is given by

(1) $$\begin{array}{r} C_{\text{m}}\frac{dV_{j}}{dt}=-g_{\text{L}}\left(V_{j}-E_{\text{L}}\right)+g_{\text{L}}\Delta_{\text{T}}\exp\left(\frac{V_{j}-V_{\text{T}}}{\Delta_{\text{T}}}\right)\\ \;-w_{j}+I+I_{j}^{\text{chem}},\\ \tau_{w}\frac{dw_{j}}{dt}=a_{j}\left(V_{j}-E_{\text{L}}\right)-w_{j},\\ \tau_{\text{s}}\frac{dg_{j}}{dt}=-g_{j}. \end{array}$$

The membrane potential *V*_*j*_ and adaptation current *w*_*j*_ represent the state of each neuron *j*. The capacitance membrane is set to *C*_m_ = 200 pF, the leak conductance to *g*_L_ = 12 nS, the leak reversal potential to *E*_L_ = −70 mV, the slope factor to Δ_T_ = 2 mV, and the spike threshold to *V*_T_ = −50 mV. We consider the injection of current *I* = 270 pA, which is the intensity above the rheobase current. The application of this constant current allows that the neurons change their potentials from resting potentials to spikes. The level of the subthreshold and triggered adaptation are represented by *a*_*j*_ and *b*_*j*_, respectively. We consider inhibitory neurons of fast spiking activities without adaptation (*a*_*j*_ = 0 and *b*_*j*_ = 0) and excitatory neurons of regular spiking with adaptation mechanisms (*a*_*j*_ = [1.9, 2.1] nS and *b*_*j*_ = 70 pA). Neuronal adaptation corresponds to the capacity of the neuronal membrane in adapting to its excitability according to the past neuronal activity. A sub- and a triggered-threshold adaptation mechanism can be associate with the parameters *a*_*j*_ and *b*_*j*_, respectively. The adaptation current also depends on the adaptation time constant τ_*w*_ = 300 ms. *g*_*j*_ represents the synaptic conductance of each neuron *j* with an exponential decay associated with the synaptic time constant τ_s_ = 2.728 ms. The connections from excitatory and inhibitory neurons are related to the excitatory and inhibitory matrix, ${\vec{M}}_{\text{exc}}$ and ${\vec{M}}_{\text{inh}}$, where each matrix element is identified as $M_{jk}^{\text{exc}}$ and $M_{jk}^{\text{inh}}$, respectively. A matrix element is equal to 1 when there is a connection from *k* to *j* or 0 in the absence of a connection. The excitatory and inhibitory elements of the matrix are associated with the red and blue dots in Figure 1. The chemical current input $I_{j}^{\text{chem}}$ arriving on each neuron *j* is defined by the expression

(2) $$\begin{array}{r} I_{j}^{\text{chem}}=I_{j}^{\text{exc}}+I_{j}^{\text{inh}} \end{array}$$

where the excitatory and inhibitory currents are given by

(3) $$\begin{array}{r} I_{j}^{\text{exc}}=I_{j}^{\text{ee}}+I_{j}^{\text{ei}}+I_{j}^{\text{ee},\text{A}}+I_{j}^{\text{ei},\text{A}},\\ \;=\left[V_{\text{REV}}^{\text{exc}}-V_{j}\right]\sum_{k=1}^{N_{\text{T}}}M_{jk}^{\text{exc}}g_{k}\left(t-d_{\text{exc}}\right), \end{array}$$

and

(4) $$\begin{array}{r} I_{j}^{\text{inh}}=I_{j}^{\text{ii}}+I_{j}^{\text{ie}}+I_{j}^{\text{ii},\text{A}}+I_{j}^{\text{ie},\text{A}}\\ \;=\left[V_{\text{REV}}^{\text{inh}}-V_{j}\right]\overset{N_{\text{T}}}{\underset{k=1}{\sum }}M_{jk}^{\text{inh}}g_{k}\left(t-d_{\text{inh}}\right), \end{array}$$

where $I_{j}^{\text{xy}}$ and $I_{j}^{\text{xy},\text{A}}$ are associated with the excitatory (x=e) or inhibitory connectivity (x=i) arriving at the excitatory (y=e) or inhibitory neurons (y=i). The type of synapse depends on the synaptic reversal potential *V*_REV_. We consider the $V_{\text{REV}}^{\text{exc}}=0$ mV for excitatory and $V_{\text{REV}}^{\text{inh}}=-80$ mV for inhibitory synapses. *N*_T_ is the total number of neurons in the network. When the membrane potential of the neuron *j* is above the threshold *V*_*j*_ > *V*_thres_ (Naud et al., 2008), the state variable is updated by the rule

(5) $$\begin{array}{r} V_{j}\to V_{\text{r}},\\ w_{j}\to w_{j}+b_{j},\\ g_{j}\to g_{j}+g_{\text{s}}. \end{array}$$

In our simulations, we consider *V*_r_ = −58 mV. The value of *b*_*j*_ depends whether the neuron *j* is excitatory or inhibitory. Each synaptic current is related to the respective conductance *g*_s_. Inside of each area, *g*_s_ is equal to *g*_ee_ for synapses between excitatory neurons, *g*_ei_ for synapses from excitatory to inhibitory neurons, *g*_ii_ for synapses between inhibitory neurons, and *g*_ie_ for synapses from inhibitory to excitatory neurons. Between different areas we include the superscript “A.” The time delay in the conductance is *d*_exc_ = 1.5 ms for excitatory connections and *d*_inh_ = 0.8 ms for inhibitory ones (Borges et al., 2020).

Table 1 gives the values of the parameters used in our simulations. We consider *g*_ee_ = 0.5 nS, *g*_ii_ = 2 nS, and *g*_ie_ = 1.5 nS. The areas exhibit synchronous and desynchronous behaviour when uncoupled between them for *g*_ei_ = 1 nS and *g*_ei_ = 2 nS, respectively. The area 1 is considered synchronised and desynchronised when uncoupled, while the area 2 is always considered desynchronised when uncoupled.

**Table 1:** Values of the parameters, where the excitatory values are indicated by • and the inhibitory ones by ⋆.

**Parameter**	**Description**	**Value**
*N*	AEIFs in each area	1,000 neurons
Areas	Number of areas	2
*A*	Area number	1 or 2
*N*_T_	Total number of neurons	2,000 neurons
*C*_m_	Capacitance membrane	200 pF
*g*_L_	Leak conductance	12 nS
*E*_L_	Leak reversal potential	–70 mV
*I*	Constant input current	270 pA
Δ_T_	Slope factor	2 mV
*V*_T_	Threshold potential	–50 mV
τ_*w*_	Adaptation time constant	300 ms
*V*_r_	Reset potential	–58 mV
*M*_*ij*_	Adjacent matrix elements	0 or 1
τ_s_	Synaptic time constant	2.728 ms
*t*_fin_	Final time to analyses	100 s
*t*_ini_	Initial time to analyses	20 s
*a*_*i*_	Subthreshold adaptation	[1.9, 2.1] nS ∙
		0 nS ⋆
*b*_*j*_	Triggered adaptation	70 pA ∙
		0 pA ⋆
*V*_REV_	Synaptic reversal potential	$V_{\text{REV}}^{\text{exc}}$ = 0 mV ∙
		$V_{\text{REV}}^{\text{inh}}$ = -80 mV ⋆
*g*_s_	Synaptic conductances	*g*_ee_, *g*_ei_, $g_{\text{ei}}^{\text{A}}$, $g_{\text{ee}}^{\text{A}}$ ∙
		*g*_ii_, *g*_ie_, $g_{\text{ii}}^{\text{A}}$, $g_{\text{ie}}^{\text{A}}$ ⋆
*g*_ee_	Excitatory to excitatory ⊙	0.5 nS ∙
*g*_ei_	Excitatory to inhibitory ⊙	1 or 2 nS ∙
*g*_ii_	Inhibitory to inhibitory ⊙	2 nS ⋆
*g*_ie_	Inhibitory to excitatory ⊙	1.5 nS ⋆
$g_{\text{ee}}^{\text{A}}$	Excitatory to excitatory ⊕	[0,3] nS ∙
$g_{\text{ei}}^{\text{A}}$	Excitatory to inhibitory ⊕	[0,6] nS ∙
$g_{\text{ii}}^{\text{A}}$	Inhibitory to inhibitory ⊕	[0,4] nS ⋆
$g_{\text{ie}}^{\text{A}}$	Inhibitory to excitatory ⊕	[0,4] nS ⋆
*d*_*j*_	Time delay	*d*_exc_ = 1.5 ms ∙
		*d*_inh_ = 0.8 ms ⋆

*The connections inside the area are identified by ⊙ and between the areas by ⊕*.

The initial values of *V*_*j*_ are randomly distributed in the interval [−70, −50] mV for all neurons. The initial values of *w*_*j*_ are randomly distributed in the interval [0, 300] pA for excitatory neurons and equal to 0 for inhibitory ones. The initial value of *g*_*j*_ is equal to 0 for all neurons. To solve the delayed differential equations, we consider that the excitatory and inhibitory neurons in the network are not spiking before the beginning of the simulation (*t* = 0). To integrate the set of ordinary differential equations, we use the 4th Runge-Kutta method with the time-step of integration equal to 10^−2^ ms.

### 2.3. Synchronisation and Relative Phase Angle

The synchronous behaviour in the network can be identified by means of the complex phase order parameter (Kuramoto, 1984)

(6) $$\begin{array}{r} R\left(t\right)=|\frac{1}{N_{\text{T}}}\overset{N_{\text{T}}}{\underset{j=1}{\sum }}\exp\left[\text{i}\psi_{j}\left(t\right)\right]|, \end{array}$$

where *R*(*t*) is the amplitude of a centroid phase vector over time. The phase of each neuron *j* is obtained through

(7) $$\begin{array}{r} \psi_{j}\left(t\right)=2\pi m+2\pi \frac{t-t_{j,m}}{t_{j,m+1}-t_{j,m}}, \end{array}$$

where *t*_*j, m*_ corresponds to the time of the *m*−th spike of the neuron *j* (*t*_*j,m*_ < *t* < *t*_*j, m*+1_) (Rosenblum et al., 1997). We consider that the spike occurs when *V*_*j*_ > *V*_thres_. The value of *R*(*t*) is equal to 0 for completely desynchronised behaviour and equal to 1 for fully synchronised patterns.

We calculate the time-average order parameter $\bar{R}$ (Batista et al., 2017)

(8) $$\begin{array}{r} \bar{R}=\frac{1}{t_{\text{fin}}-t_{\text{ini}}}\overset{t_{\text{fin}}}{\underset{t_{\text{ini}}}{\int }}R\left(t\right)dt, \end{array}$$

where *t*_fin_−*t*_ini_ is the time window with *t*_fin_ = 100 s and *t*_ini_ = 20 s.

The order parameter for each area is given by

(9) $$\begin{array}{r} R_{A}\left(t\right)=|\frac{1}{N}\overset{A\cdot N}{\underset{j=\left(A-1\right)\cdot N+1}{\sum }}\exp\left[\text{i}\psi_{j}\left(t\right)\right]|, \end{array}$$

where *A* denotes the area number. The mean value of *R*_*A*_(*t*) (${\bar{R}}_{A}$) is computed by Equation (8). The resultant phase angle of each area *A* is defined as

(10) $$\begin{array}{r} \Theta_{A}\left(t\right)=\arctan\left(\frac{R_{A}^{\text{y}}\left(t\right)}{R_{A}^{\text{x}}\left(t\right)}\right). \end{array}$$

The real $R_{A}^{\text{x}}$ and complex $R_{A}^{\text{y}}$ components of the order parameter can be described as

(11) $$\begin{array}{r} R_{A}^{\text{x}}\left(t\right)=\frac{1}{N}\sum_{j=\left(A-1\right)\cdot N+1}^{A\cdot N}\cos\left[\psi_{j}\left(t\right)\right], \end{array}$$

and

(12) $$\begin{array}{r} R_{A}^{\text{y}}\left(t\right)=\frac{1}{N}\sum_{j=\left(A-1\right)\cdot N+1}^{A\cdot N}\sin\left[\psi_{j}\left(t\right)\right], \end{array}$$

respectively. Θ_*A*_(*t*) evolves in the counter-clockwise direction, since each individual neuron evolves in this direction.

We define a relative phase angle for each area $\Theta_{A}^{\prime }\left(t\right)$ (Varela et al., 2001) as

(13) $$\begin{array}{r} \Theta_{A}^{\prime }\left(t\right)=\Theta_{A}\left(t\right)-\Theta_{1}\left(t\right). \end{array}$$

The phase of the area 1 changes over time and its relative value $\Theta_{1}^{\prime }\left(t\right)$ is equal to 0. For the area 2, the relative phase angle $\Theta_{2}^{{}^{\prime }}\left(t\right)$ can change over time. We calculate the mean value of the relative phase angle of the area 2 by means of

(14) $$\begin{array}{r} \bar{\Theta_{2}^{\prime }}=\frac{1}{t_{\text{fin}}-t_{\text{ini}}}\int_{t_{\text{ini}}}^{t_{\text{fin}}}\Theta_{2}^{\prime }\left(t\right)dt. \end{array}$$

To compute the predominant rotation direction of the area 2, we consider the first-order derivative of their relative phase angle, which corresponds to the instantaneous velocity of the relative phase angle (rad/s), that is given by

(15) $$\begin{array}{r} {\overset{∙}{\Theta }}_{2}^{\prime }\left(t\right)=\frac{d\Theta_{2}^{\prime }\left(t\right)}{dt}. \end{array}$$

The mean value of the relative velocity of the area 2 is obtained via

(16) $$\begin{array}{r} \bar{{\overset{∙}{\Theta }}_{2}^{\prime }}=\frac{1}{t_{\text{fin}}-t_{\text{ini}}}\int_{t_{\text{ini}}}^{t_{\text{fin}}}{\overset{∙}{\Theta }}_{2}^{\prime }\left(t\right)dt. \end{array}$$

The value is close to 0 for non-preponderant direction of rotation and positive (negative) for predominant counter-clockwise (clockwise) direction.

The variability of $\Theta_{2}^{\prime }$ is given by

(17) $$\sigma_{\Theta_{2}^{\prime }}=\sqrt{\bar{\Theta_{2}^{\prime }{}^{2}}-{\bar{\Theta_{2}^{\prime }}}^{2}}.$$

**Figure 2:**
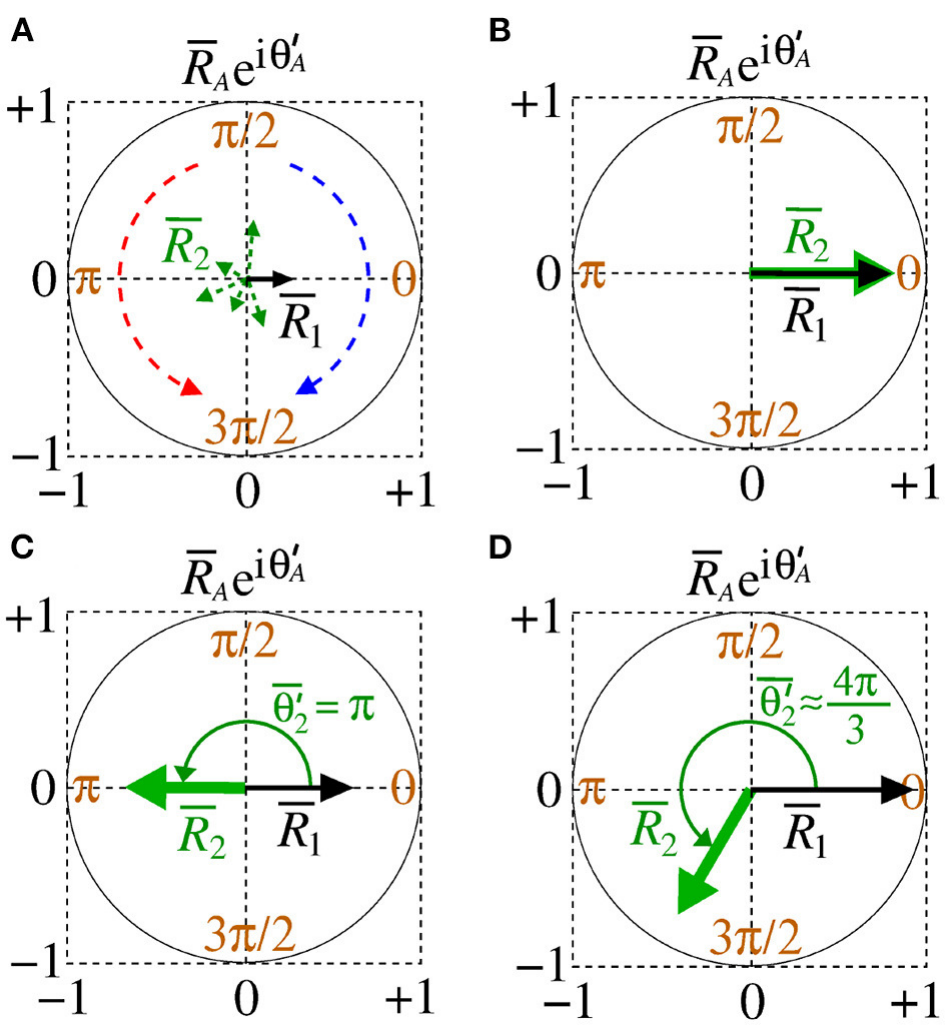
Schematic representation of the mean order parameter of the area 1 and area 2 for **(A)** out-of-phase, **(B)** phase, **(C)** anti-phase, and **(D)** shift-phase synchronisation.

Small and high deviations are given by $\sigma_{\Theta_{A}^{\prime }}\approx 0$ and $\sigma_{\Theta_{A}^{\prime }}>0.5$, respectively. Figure 2 shows a schematic representation of the mean order parameter of the area 1 and area 2 for Figure 2A desynchronised patterns of both areas out-of-phase, Figure 2B in-phase ($\bar{\Theta_{2}^{\prime }}=0$), Figure 2C anti-phase ($\bar{\Theta_{2}^{\prime }}=\pi$), and Figure 2D shift-phase synchronisation ($\bar{\Theta_{2}^{\prime }}\approx 4\pi /3$). In Figure 2A, the clockwise (blue) and counter-clockwise (red) arrows indicate that the relative phase angle of area 2 change over time. The amplitude and direction of the relative phase angle of the area *A* can be described by ${\bar{R}}_{A}{\text{e}}^{\text{i}\Theta_{A}^{\prime }}$. In our simulations, we observe results in which desynchronised activities can be related to high variability of the relative phase angle of the area 2. When the areas are synchronised, the variability of the relative phase angle can go to 0. The mean value of relative phase angle is efficient for small variability of the relative phase angle.

## 3. Unidirectional Interaction Between the Areas

### 3.1. Excitatory Connections

**Figure 3:**
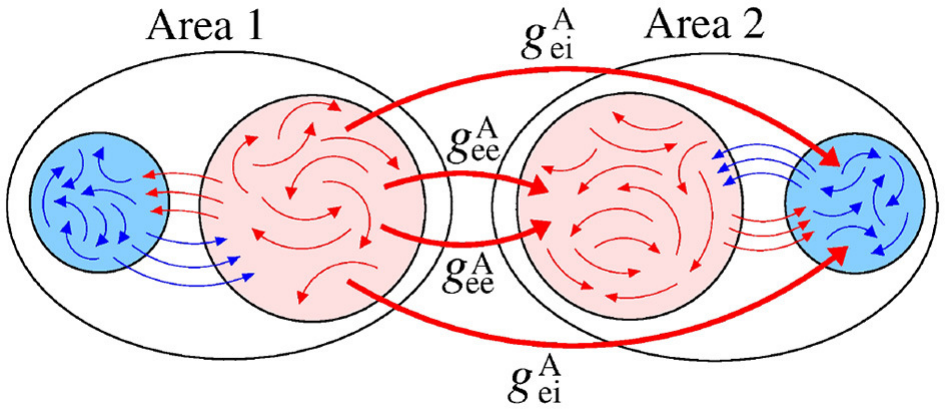
Schematic representation of the excitatory connections (red arrows) from the area 1 (sender) to the neurons in the area 2 (receiver). The red and blue arrows represent the excitatory and inhibitory connections, respectively.

We analyse a neuronal network separated into two areas (sender-receiver) coupled by means of the excitatory connections. Figure 3 displays a schematic representation of the sender area 1 to receiver area 2 via excitatory connections, that are related to the $g_{\text{ei}}^{\text{A}}$ and $g_{\text{ee}}^{\text{A}}$ conductances.

Firstly, we consider the case in which the neurons in the sender and receiver area are desynchronised. Through the time evolution of $\Theta_{2}^{\prime }$, we verify that, depending on the conductance values, it can occur a relative counter-clockwise rotation, clockwise rotation, or neither of them in the area 2. We observe that $g_{\text{ee}}^{\text{A}}$ contributes to generate positive $\bar{{\overset{∙}{\Theta }}_{2}^{\prime }}$, while $g_{\text{ei}}^{\text{A}}$ to negative one. Both areas show order parameters with small values, i.e., the neurons remain desynchronised.

**Figure 4:**
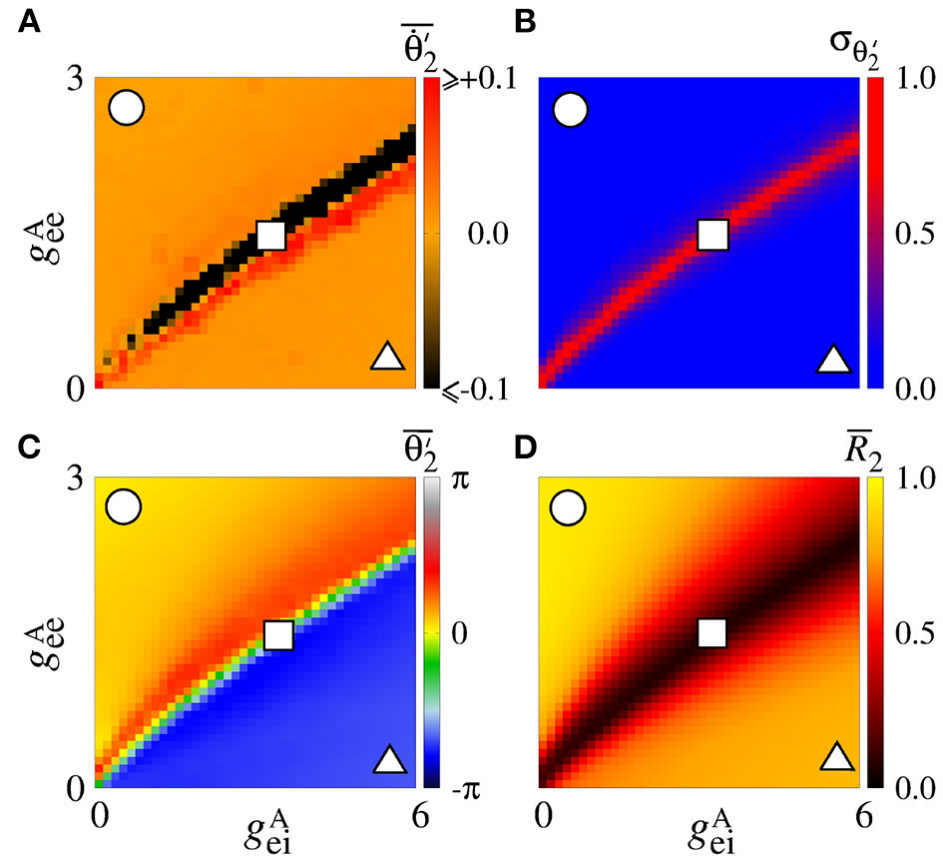
Unidirectional excitatory interaction between synchronised neurons in the sender area and desynchronised neurons in the receiver area. Synchronised sender area can generate phase and shift-phase synchronisation due to $g_{\text{ee}}^{\text{A}}$ and $g_{\text{ei}}^{\text{A}}$, respectively. **(A)** The predominant direction of rotation of the relative phase angle of the area 2, **(B)** the standard deviation of the relative phase angle of the area 2, **(C)** the mean relative phase angle of the area 2, and **(D)** the mean order parameter of the area 2. The circle, square, and triangle symbols indicate $g_{\text{ee}}^{\text{A}}=2.7$ nS and $g_{\text{ei}}^{\text{A}}=0.6$ nS, $g_{\text{ee}}^{\text{A}}=1.5$ nS and $g_{\text{ei}}^{\text{A}}=3$ nS, and $g_{\text{ee}}^{\text{A}}=0.3$ nS and $g_{\text{ei}}^{\text{A}}=5.4$ nS, respectively.

Secondly, we consider that the neurons in the sender area are synchronised while the neurons in the receiver area are initially desynchronised. In Figure 4A, the parameter space $g_{\text{ee}}^{\text{A}}\times g_{\text{ei}}^{\text{A}}$ exhibits values of $\bar{{\overset{∙}{\Theta }}_{2}^{\prime }}$ in [−0.1, 0.1] rad/s, where the $\bar{{\overset{∙}{\Theta }}_{2}^{\prime }}$ values approximately or less than −0.1 are in a small black diagonal region. For a large set of parameters, $\bar{{\overset{∙}{\Theta }}_{2}^{\prime }}$ is close to zero. Figure 4B displays $\sigma_{\Theta_{2}^{\prime }}$ values about 0, except for a small red region where the values are greater than 0.5. We compute the mean relative phase angle of the area 2, as shown in Figure 4C. We verify that $g_{\text{ee}}^{\text{A}}$ leads the area 2 to a value of the phase angle equal to 0, while $g_{\text{ei}}^{\text{A}}$ leads to a shifted phase angle, $\bar{\Theta_{2}^{{}^{\prime }}}\approx 4\pi /3$. The stabilisation of the phase angle is associated with the synchronisation of the area 2, as shown in Figure 4D. We see a large region in which ${\bar{R}}_{2}$ is close to 1, meaning that the neurons in the area 2 are synchronised.

**Figure 5:**
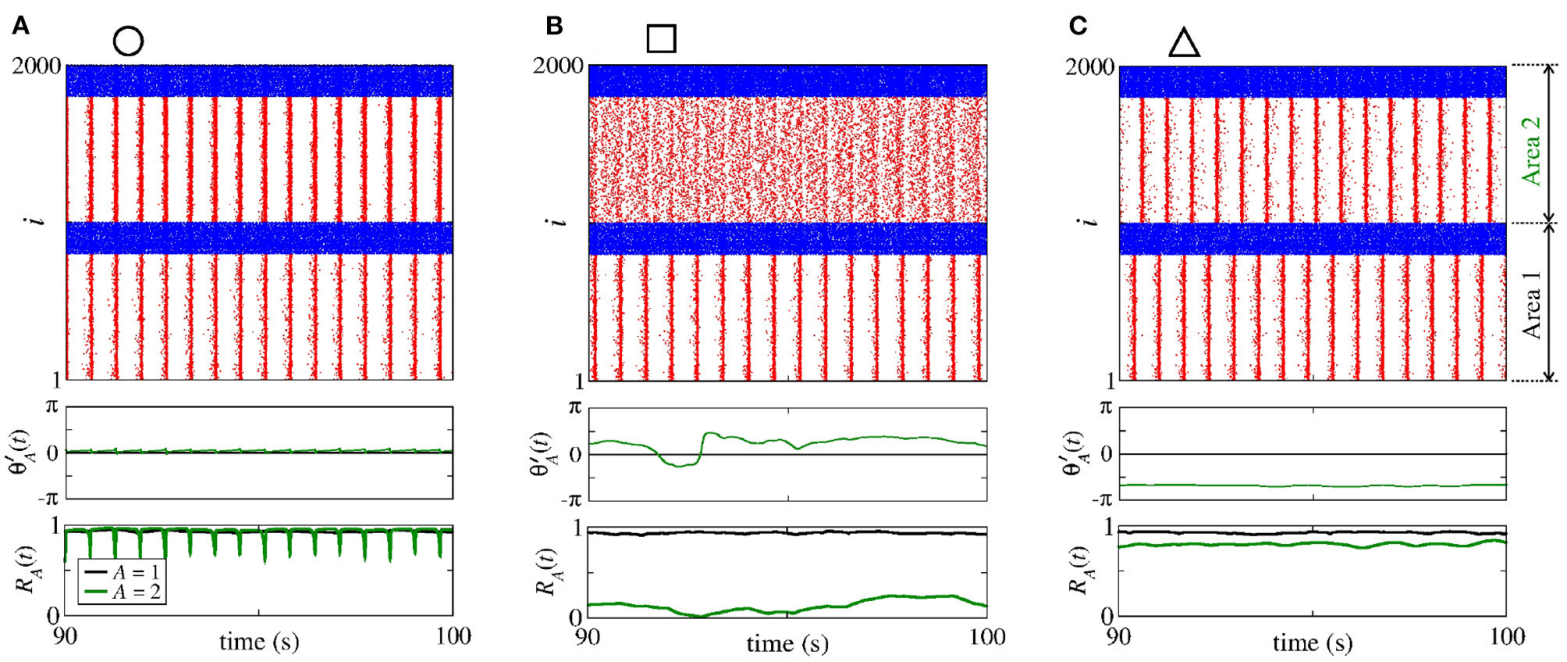
Raster plot (top), time evolution of $\Theta_{2}^{\prime }$ (middle), and time evolution of the order parameter (bottom) for **(A)** $g_{\text{ee}}^{\text{A}}=2.7$ nS and $g_{\text{ei}}^{\text{A}}=0.6$ nS, **(B)** $g_{\text{ee}}^{\text{A}}=1.5$ nS and $g_{\text{ei}}^{\text{A}}=3$ nS, and **(C)** $g_{\text{ee}}^{\text{A}}=0.3$ nS and $g_{\text{ei}}^{\text{A}}=5.4$ nS. We consider unidirectional interactions with excitatory connections between the areas, where the neurons are synchronised in the sender area and the neurons are initially desynchronised in the receiver area. In the raster plots, the blue and red dots indicate the fires of the inhibitory and excitatory neurons, respectively. Synchronised sender area can actuate on the initially desynchronised receiver area generating **(A)** phase synchronisation due to $g_{\text{ee}}^{\text{A}}$, **(B)** desynchronisation due to both $g_{\text{ee}}^{\text{A}}$ and $g_{\text{ei}}^{\text{A}}$, and **(C)** shift-phase synchronisation due to $g_{\text{ei}}^{\text{A}}$. In **(A)**, *R*_1_ (black line) and *R*_2_ (green line) exhibit different values due to the fact that there are some neurons with bursting activity in the area 2. The bursts are generated due to the excitatory connections from the area 1 to the excitatory neurons of the area 2. In **(C)**, *R*_2_ is smaller than *R*_1_ due to the excitatory connections from the area 1 to the inhibitory neurons of the area 2.

Figure 5 displays the raster plot (top), relative phase angle (middle), and order parameter (bottom) for (a) $g_{\text{ee}}^{\text{A}}=2.7$ nS and $g_{\text{ei}}^{\text{A}}=0.6$ nS, (b) $g_{\text{ee}}^{\text{A}}=1.5$ nS and $g_{\text{ei}}^{\text{A}}=3$ nS, and (c) $g_{\text{ee}}^{\text{A}}=0.3$ nS and $g_{\text{ei}}^{\text{A}}=5.4$ nS, that are indicated in Figure 4 through the circle, square, and triangle symbols, respectively. In Figure 5A, we see that $\Theta_{2}^{\prime }$ is closed to the sender phase angle and the receiver area has synchronised neurons due to a high $g_{\text{ee}}^{\text{A}}$ conductance. Figure 5B shows that for a combination of $g_{\text{ee}}^{\text{A}}$ and $g_{\text{ei}}^{\text{A}}$, neurons in the area 2 are not synchronised. In Figure 5C, we observe that due to high $g_{\text{ei}}^{\text{A}}$ conductance, neurons in the area 2 have a shift-phase synchronisation, corresponding to $\bar{\Theta_{2}^{{}^{\prime }}}\approx 4\pi /3$, as indicated in Figure 2D.

### 3.2. Inhibitory Connections

**Figure 6:**
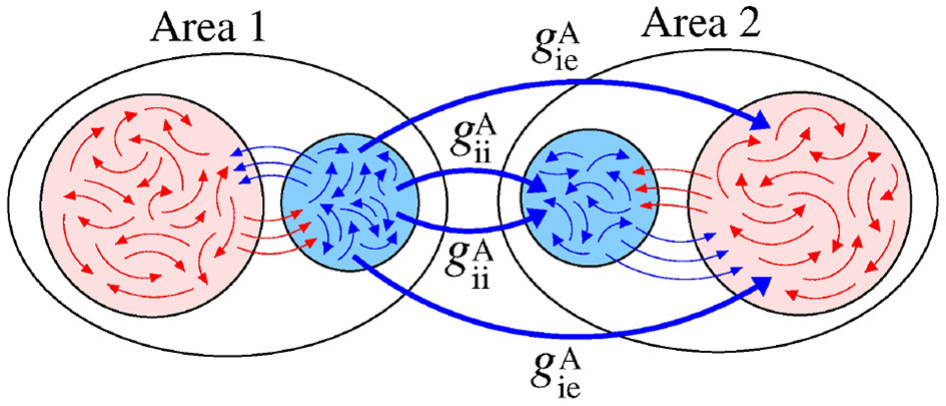
Schematic representation of inhibitory connections from the area 1 to the neurons in the area 2.

Figure 6 displays a schematic representation of the sender area to the receiver area via the $g_{\text{ii}}^{\text{A}}$ and $g_{\text{ie}}^{\text{A}}$ conductances.

**Figure 7:**
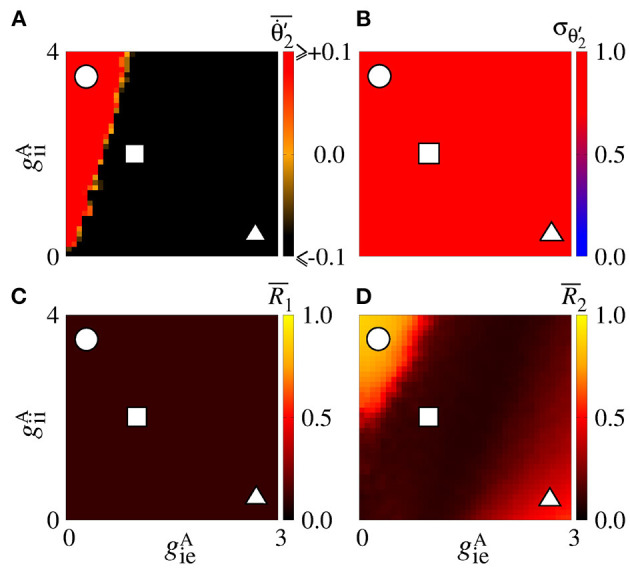
Unidirectional inhibitory interaction between two coupled areas with desynchronised neurons. Desynchronised sender area can generate synchronisation and silence on excitatory neurons of the receiver area due to $g_{\text{ii}}^{\text{A}}$ and $g_{\text{ie}}^{\text{A}}$, respectively. **(A)** The predominant direction of rotation of the relative phase angle of the area 2, **(B)** the standard deviation of the relative phase angle of the area 2, **(C)** the mean order parameter of the area 1, and **(D)** the mean order parameter of the area 2. The circle, square, and triangle symbols indicate $g_{\text{ii}}^{\text{A}}=3.6$ nS and $g_{\text{ie}}^{\text{A}}=0.3$ nS, $g_{\text{ii}}^{\text{A}}=2$ nS and $g_{\text{ie}}^{\text{A}}=1$ nS, and $g_{\text{ii}}^{\text{A}}=0.4$ nS and $g_{\text{ie}}^{\text{A}}=2.7$ nS, respectively.

For desynchronised neurons in the sender and receiver areas, increasing $g_{\text{ii}}^{\text{A}}$, we see that $\bar{{\overset{∙}{\Theta }}_{2}^{\prime }}$ is positive for small values of $g_{\text{ie}}^{\text{A}}$, as shown in Figure 7A. On the other hand, $g_{\text{ie}}^{\text{A}}$ contribute to negative values of $\bar{{\overset{∙}{\Theta }}_{2}^{\prime }}$. Figure 7B exhibits a high variability ($\sigma_{\Theta_{2}^{{}^{\prime }}}>0.5$) of $\Theta_{2}^{{}^{\prime }}$. In Figures 7C,D, we compute the mean order parameters for the areas 1 and 2, respectively. The neurons are desynchronised in the area 1, while in the area 2, we see a small region in the parameter space where there is synchronous behaviour (${\bar{R}}_{2}>0.8$). The increase of ${\bar{R}}_{2}$ is due to the $g_{\text{ii}}^{\text{A}}$ parameter, that induces the inhibition of the inhibitory neurons of the receiver area (circle). For high $g_{\text{ie}}^{\text{A}}$ values (triangle), a large number of excitatory neurons do not fire. A similar result was reported by Zhou et al. (2010), where silent activities of excitatory neurons were observed due to a strong inhibition. Urban-Ciecko et al. (2015) found which inhibition can silence excitatory synapses in the neocortex. Pals et al. (2020) demonstrated that activity-silence maintenance can be related to a working memory process. The silence of neurons has received great attention in the last years (Mochol et al., 2015; Wiegert et al., 2015; Barbosa et al., 2020; Xu et al., 2020).

**Figure 8:**
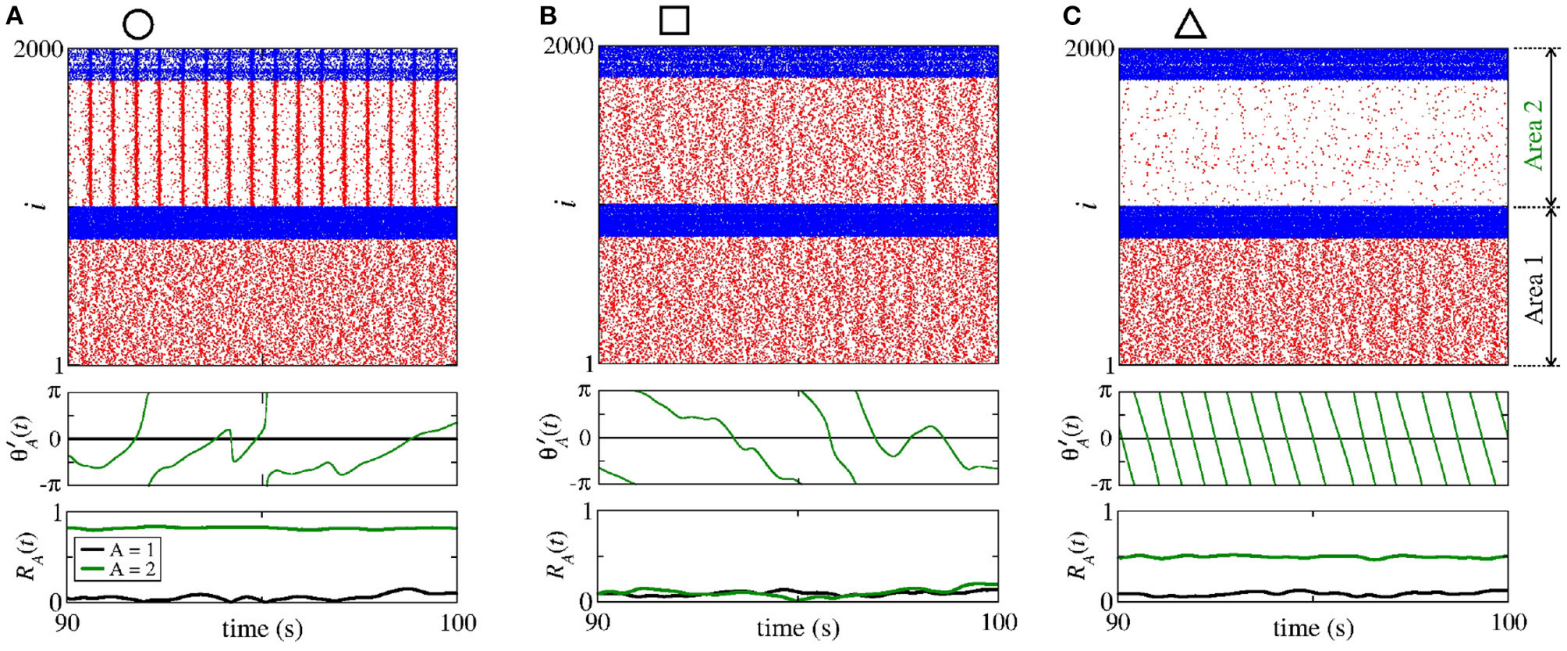
Raster plot (top), time evolution of $\Theta_{A}^{\prime }$ (middle), and time evolution of the order parameter (bottom) for **(A)** $g_{\text{ii}}^{\text{A}}=3.6$ nS and $g_{\text{ie}}^{\text{A}}=0.3$ nS, **(B)** $g_{\text{ii}}^{\text{A}}=2$ nS and $g_{\text{ie}}^{\text{A}}=1$ nS, and **(C)** $g_{\text{ii}}^{\text{A}}=0.4$ nS and $g_{\text{ie}}^{\text{A}}=2.7$ nS. We consider unidirectional interaction with inhibitory connections between the areas, where the neurons are desynchronised in the sender area and the neurons are initially desynchronised in the receiver area. *A* = 1 and *A* = 2 in black and green lines, respectively. Desynchronised sender area can actuate on the initially desynchronised receiver area generating **(A)** synchronisation due to $g_{\text{ii}}^{\text{A}}$, **(B)** desynchronisation due to both $g_{\text{ii}}^{\text{A}}$ and $g_{\text{ie}}^{\text{A}}$, and **(C)** silent activities of excitatory neurons due to $g_{\text{ie}}^{\text{A}}$ conductances.

Figure 8 displays the raster plot (top), time evolution of $\Theta_{2}^{\prime }$ (middle), and time evolution of the order parameter (bottom) for the values of $g_{\text{ii}}^{\text{A}}$ and $g_{\text{ie}}^{\text{A}}$ indicated by circle, square, and triangle symbols in Figure 7. For $g_{\text{ii}}^{\text{A}}=3.6$ nS and $g_{\text{ie}}^{\text{A}}=0.3$ nS (Figure 8A), the neurons in the area 2 synchronise and $\Theta_{2}^{{}^{\prime }}$ denotes a relative counter-clockwise rotation. For $g_{\text{ii}}^{\text{A}}=2$ nS and $g_{\text{ie}}^{\text{A}}=1$ nS (Figure 8B), the neurons in the area 2 are desynchronised and $\Theta_{2}^{{}^{\prime }}$ denotes a relative clockwise rotation. In Figure 8C, there is no synchronous behaviour and we see a large number of excitatory neurons that remain in silence for a long time.

**Figure 9:**
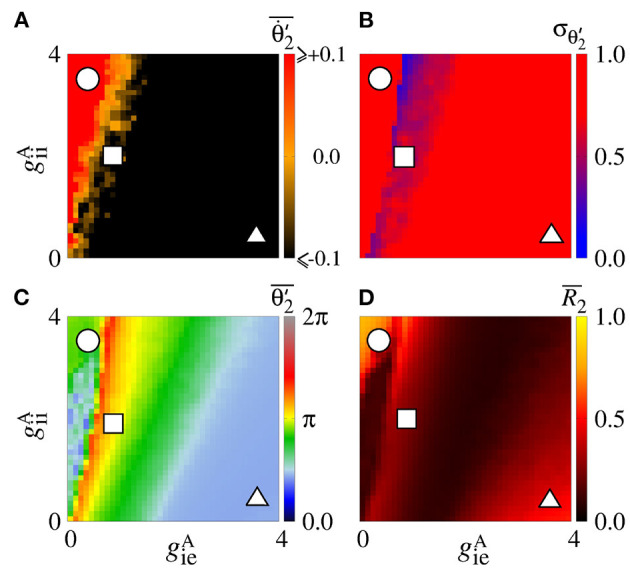
Unidirectional inhibitory interaction between two coupled areas, where the neurons in the sender area are synchronised and the neurons in the receiver area are initially desynchronised. **(A)** The predominant direction of rotation of the relative phase angle of the area 2, **(B)** the standard deviation of the relative phase angle of the area 2, **(C)** the mean relative phase angle of the area 2, **(D)** the mean order parameter of the area 2. The circle, square, and triangle symbols indicate $g_{\text{ii}}^{\text{A}}=3.6$ nS and $g_{\text{ie}}^{\text{A}}=0.4$ nS, $g_{\text{ii}}^{\text{A}}=2$ nS and $g_{\text{ie}}^{\text{A}}=0.9$ nS, and $g_{\text{ii}}^{\text{A}}=0.4$ nS and $g_{\text{ie}}^{\text{A}}=3.6$ nS, respectively. Synchronised sender area can generate synchronisation and silent activities of excitatory neurons of the receiver area depending on $g_{\text{ii}}^{\text{A}}$ and $g_{\text{ie}}^{\text{A}}$.

We also consider the case in which the sender area has synchronised neurons. Figure 9A shows that the $\bar{{\overset{∙}{\Theta }}_{2}^{\prime }}$ values are positive due to the $g_{\text{ii}}^{\text{A}}$ conductance with small values of $g_{\text{ie}}^{\text{A}}$ for small values of $g_{\text{ie}}^{\text{A}}$. This result is similar to the situation in which the neurons in the sender area are desynchronised. However, due to the synchronised neurons in the area 1, we verify the existence of regions in the parameter space $g_{\text{ii}}^{\text{A}}\times g_{\text{ie}}^{\text{A}}$ with small values of the variability, as shown in Figure 9B. $\sigma_{\Theta_{2}^{{}^{\prime }}}<0.5$ corresponds to a certain stabilisation of the relative phase angle of the area 2. Figure 9C displays the mean relative phase angle of the area 2. For the lowest $\sigma_{\Theta_{2}^{{}^{\prime }}}$, we find a region where $\bar{\Theta_{2}^{\prime }}\approx \pi$. In Figure 9D, we see that synchronous behaviour in the area 2 for large $g_{\text{ii}}^{\text{A}}$ and small $g_{\text{ie}}^{\text{A}}$ values arise, where there is a circle symbol. Partial anti-phase synchronisation is observed in the region indicated by the square. The triangle denotes the region in which a high inhibition of the excitatory neurons occurs, and as a consequence a great quantity of excitatory neurons in the receiver area are silenced.

## 4. Bidirectional Interaction Between the Areas

### 4.1. Excitatory Connections

**Figure 10:**
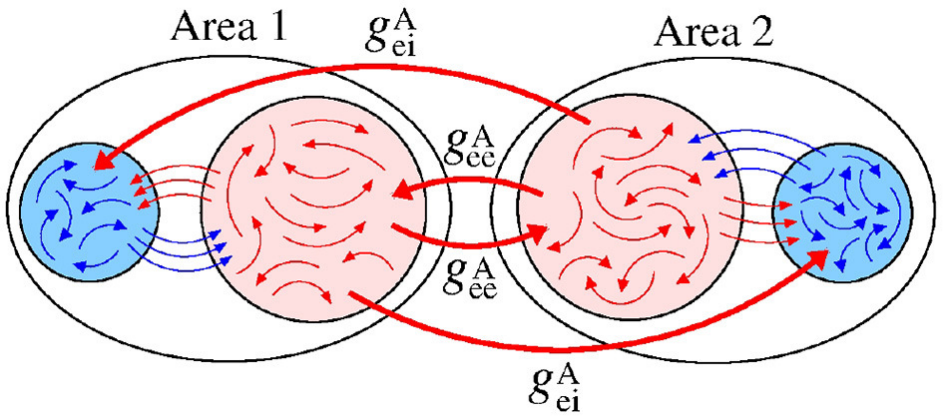
Schematic representation of bidirectional interaction between the areas by means of excitatory connections.

Figure 10 exhibits a schematic representation of bidirectional interactions via excitatory connections with $g_{\text{ei}}^{\text{A}}$ and $g_{\text{ee}}^{\text{A}}$ conductances. Without an interaction between the areas ($g_{\text{ee}}^{\text{A}}=g_{\text{ei}}^{\text{A}}=0$), the neurons exhibit desynchronised activities.

**Figure 11:**
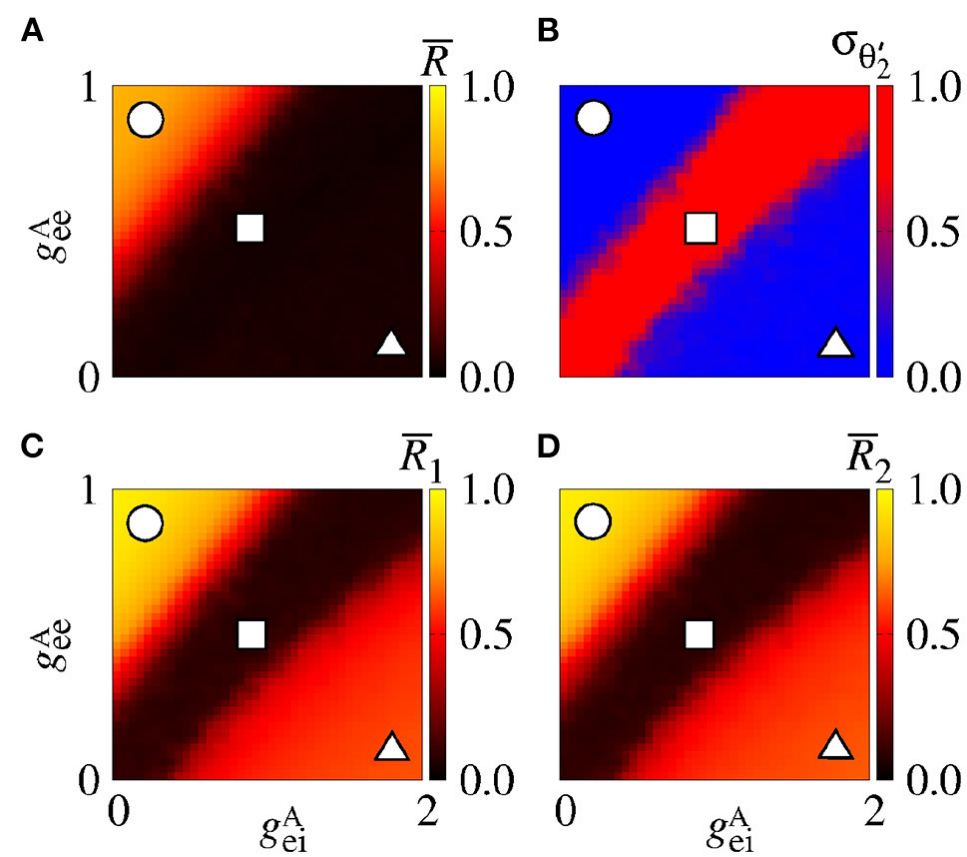
Bidirectional excitatory interaction between two areas with desynchronised neurons. **(A)** The mean order parameter of the neuronal network, **(B)** the standard deviation of the relative phase angle of the area 2, **(C)** the mean order parameter of the area 1, and **(D)** the mean order parameter of the area 2. The circle, square, and triangle symbols indicate $g_{\text{ee}}^{\text{A}}=0.9$ nS and $g_{\text{ei}}^{\text{A}}=0.2$ nS, $g_{\text{ee}}^{\text{A}}=0.5$ nS and $g_{\text{ei}}^{\text{A}}=0.9$ nS, and $g_{\text{ee}}^{\text{A}}=0.1$ nS and $g_{\text{ei}}^{\text{A}}=1.8$ nS, respectively. The bidirectional excitatory connectivity can generate phase and anti-phase synchronisation between the two areas due to $g_{\text{ee}}^{\text{A}}$ and $g_{\text{ei}}^{\text{A}}$, respectively.

Figure 11A displays the mean order parameter of the neuronal network. The region, where the circle is located, has a larger value of $\bar{R}$, due to the fact that the neurons in the two areas are synchronised, namely phase synchronisation among neurons. In Figure 11B, we verify that the reduction of the variability can be associated with the synchronised activities between the areas. Figures 11C,D shows the mean order parameters of the areas 1 and 2, respectively. We can see that the regions of the small variability of $\Theta_{2}^{\prime }$ (circle and triangle symbols) correspond to the synchronised activities. For the region with large variability values (square symbol), the neurons of the areas are desynchronised. In the region where the triangle symbol is located, there is an anti-phase synchronisation between the neurons of the areas 1 and 2.

### 4.2. Inhibitory Connections

**Figure 12:**
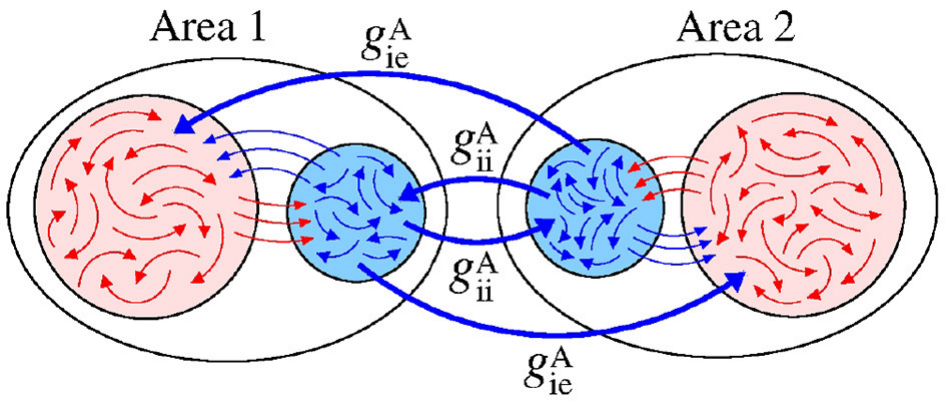
Schematic representation of bidirectional interaction between the areas by means of inhibitory connections.

Figure 12 displays a schematic representation of the bidirectional configuration interacting through inhibitory connections associated with the $g_{\text{ii}}^{\text{A}}$ and $g_{\text{ie}}^{\text{A}}$ conductances. Without interaction between the areas ($g_{\text{ii}}^{\text{A}}=g_{\text{ie}}^{\text{A}}=0$), the neurons exhibit desynchronised activities.

**Figure 13:**
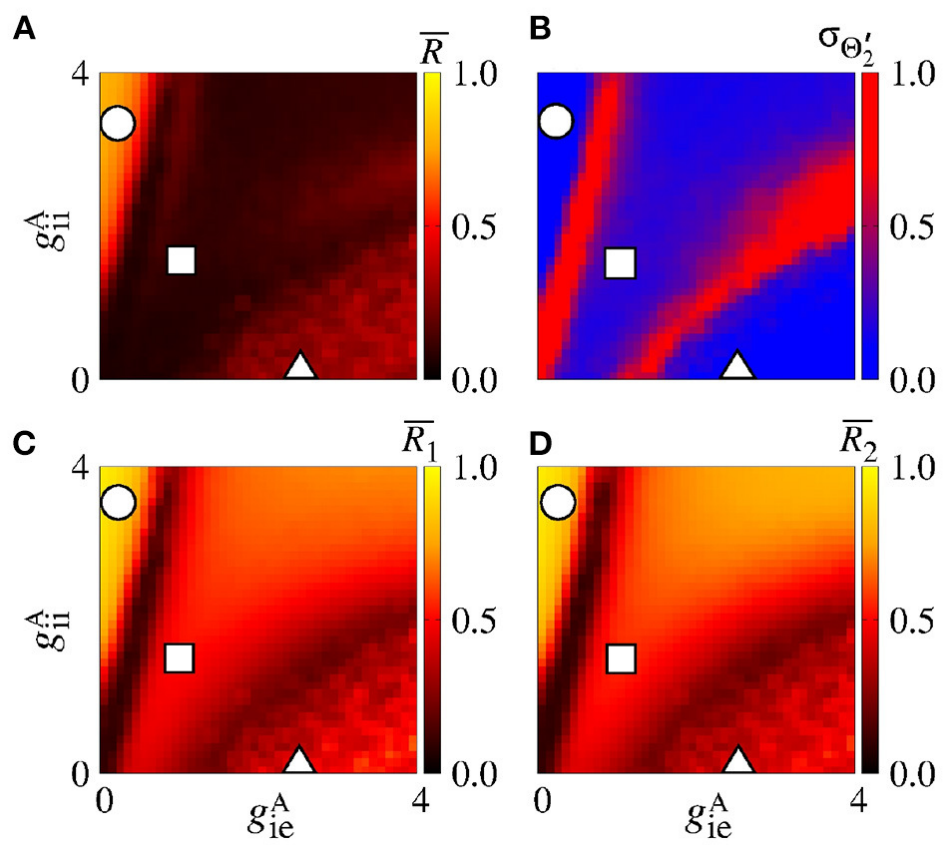
Bidirectional inhibitory interaction between two areas with desynchronised neurons. **(A)** The mean order parameter of the neuronal network, **(B)** the standard deviation of the relative phase angle of the area 2, **(C)** the mean order parameter of the area 1, and **(D)** the mean order parameter of the area 2. The circle, square, and triangle symbols indicate $g_{\text{ii}}^{\text{A}}=3.6$ nS and $g_{\text{ie}}^{\text{A}}=0.2$ nS, $g_{\text{ii}}^{\text{A}}=1.5$ nS and $g_{\text{ie}}^{\text{A}}=1$ nS, and $g_{\text{ii}}^{\text{A}}=0.2$ nS and $g_{\text{ei}}^{\text{A}}=2.5$ nS, respectively. The bidirectional inhibitory connectivity can generate phase and anti-phase synchronisation between the two areas depending on $g_{\text{ii}}^{\text{A}}$ and $g_{\text{ie}}^{\text{A}}$. In both areas, silent activities of excitatory neurons are observed for small $g_{\text{ii}}^{\text{A}}$ and large $g_{\text{ie}}^{\text{A}}$ values, respectively.

In the parameter space $g_{\text{ii}}^{\text{A}}\times g_{\text{ie}}^{\text{A}}$, the mean order parameter for the neuronal network (Figure 13A) shows a region in which the neurons in the areas are synchronised, where a circle symbol is located. For the standard deviation of relative phase angle rotation of the area 2, we identify three regions with values about 0, as shown in Figure 13B. The inhibitory connections are responsible for decreasing the relative phase angle variability (circle, square, and triangle symbols). Figures 13C,D exhibits the mean order parameter of the areas 1 and 2, respectively. The regions of small variability of $\Theta_{2}^{\prime }$ can correspond to the synchronous behaviour in each area or silence of some excitatory neurons.

**Figure 14:**
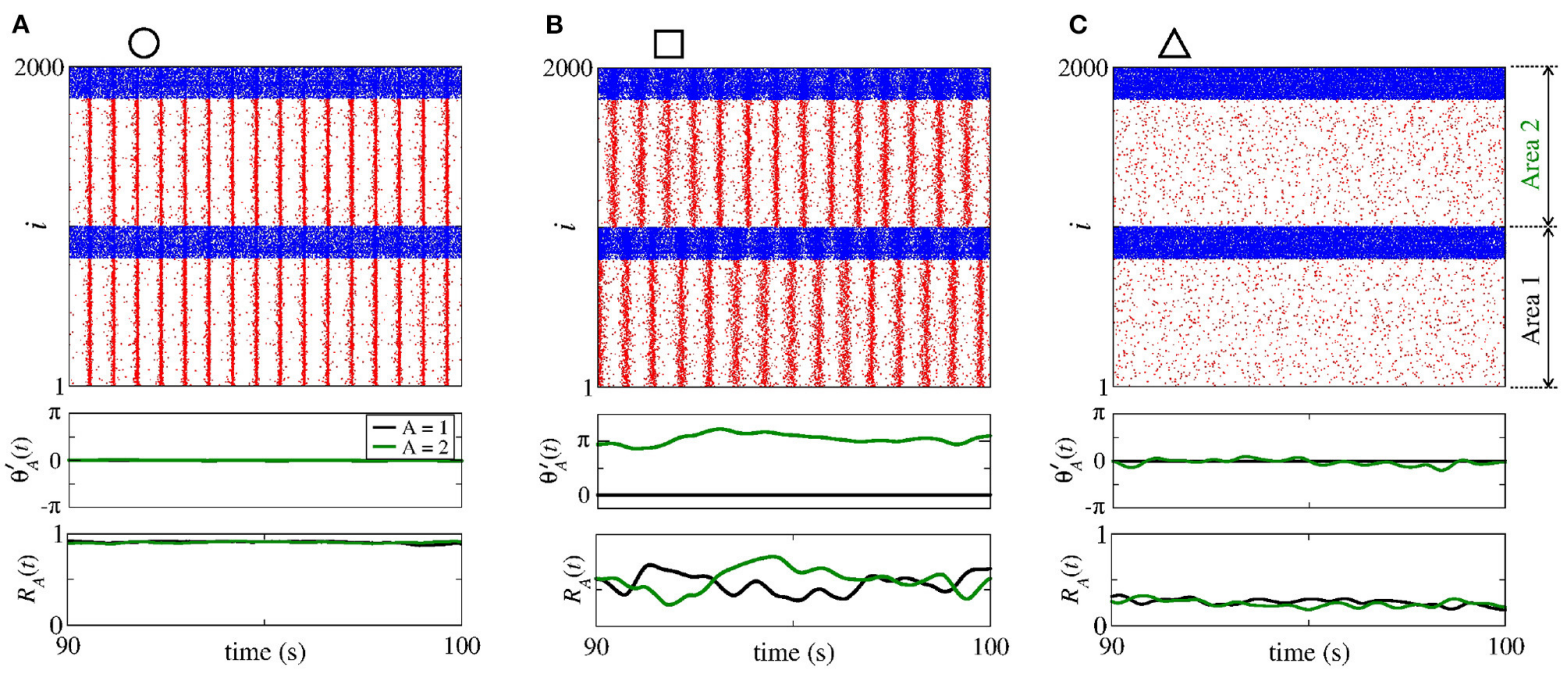
Raster plot (top), relative phase angle (middle), and order parameter (bottom) for **(A)** $g_{\text{ii}}^{\text{A}}=3.6$ nS and $g_{\text{ie}}^{\text{A}}=0.2$ nS, **(B)** $g_{\text{ii}}^{\text{A}}=1.5$ nS and $g_{\text{ie}}^{\text{A}}=1$ nS, and **(C)** $g_{\text{ii}}^{\text{A}}=0.2$ nS and $g_{\text{ei}}^{\text{A}}=2.5$ nS. We consider bidirectional interaction with inhibitory connections between the areas, where the neurons in the sender and receiver areas are initially desynchronised. Due to the bidirectional inhibitory interaction, **(A)** phase synchronisation can emerge due to $g_{\text{ii}}^{\text{A}}$, **(B)** anti-phase synchronisation due to a combination of $g_{\text{ii}}^{\text{A}}$ and $g_{\text{ie}}^{\text{A}}$, and **(C)** silent activities of excitatory neurons due to large values of $g_{\text{ie}}^{\text{A}}$.

In Figure 14, we show the raster plot (top), the relative phase angle (middle), and the order parameter (bottom) for (a) $g_{\text{ii}}^{\text{A}}=3.6$ nS and $g_{\text{ie}}^{\text{A}}=0.2$ nS, (b) $g_{\text{ii}}^{\text{A}}=1.5$ nS and $g_{\text{ie}}^{\text{A}}=1.0$ nS, and (c) $g_{\text{ii}}^{\text{A}}=0.2$ nS and $g_{\text{ei}}^{\text{A}}=2.5$ nS, according to the circle, square, and triangle symbols, respectively, denoted in Figure 13. Figure 14A displays the occurrence of phase synchronisation among the neurons between the areas. Depending on the values of $g_{\text{ii}}^{\text{A}}$ and $g_{\text{ie}}^{\text{A}}$, it is possible to observe partial anti-phase synchronisation and silenced excitatory neurons, as shown in Figures 14B,C.

## 5. Conclusions

In this work, we investigate the influence of excitatory and inhibitory connections between areas in neuronal synchronous behaviour. We build a network with two areas formed by excitatory and inhibitory neurons. The neuron dynamics is modelled by means of an adaptive exponential integrate-and-fire (AEIF) model, that is able to mimic known neuronal activities. We consider unidirectional (sender-receiver) and bidirectional interactions between the areas, as well as different coupling configurations.

In the unidirectional interaction, firstly we analyse the dynamical behaviour of the receiver area with excitatory connections from the sender area. When the neurons in the sender area are desynchronised, depending on the conductances values, counter-clockwise and clockwise rotation can arise in the receiver area. For synchronised neurons in the sender area, it is possible to observe phase and shift-phase synchronisation. Secondly, for inhibitory connections from the sender area, we find values of the conductances in which the neurons in the receiver area can be silenced, namely, they do not spike for a long time. The inhibitory connections can also induce synchronous behaviour in the neurons that belong to the receiver area even when the neurons in the sender area are desynchronised. For synchronised or desynchronised neurons in the sender area, the excitatory (inhibitory) connections to the excitatory (inhibitory) neurons in the receiver area generate an increase in the relative phase angle of the receiver area. Otherwise, excitatory (inhibitory) connections from the sender area to inhibitory (excitatory) neurons in the receiver area reduce the relative phase angle of the receiver area. We also verify that the synchronised sender area is more efficient to reduce the variability of the relative phase angle of the receiver area than the desynchronised one.

With regard to bidirectional interactions, the excitatory connections to the excitatory neurons can induce phase synchronisation, while to inhibitory neurons can favour anti-phase synchronisation. In our work, the anti-phase mechanism due to the inhibitory connections is similar to the mechanism reported by Kim and Lim (2020). They demonstrated the existence of phase-shift synchronisation among three cluster networks due to inhibitory synaptic coupling. We verify that the inhibitory connections from the areas to the inhibitory neurons of other ones can generate phase synchronisation between them due to the bidirectional interaction. In addition, when the inhibitory connections arriving at the excitatory neurons between the areas are strong, silence activities of the excitatory neurons are observed.

Our simulations suggest that the excitatory and inhibitory connections from one area to another play a crucial role in the emergence of phase, anti-phase, shift-phase synchronisation between the neurons in the areas. Our results should be useful to clarify how these types of synchronisation emerge in neuronal areas. For more than two areas, we expect to find phase synchronisation due to $g_{\text{ee}}^{\text{A}}$. Nevertheless, we believe that more complex patterns related to the synchronous behaviour will arise. In future works, we plan to study the emergence of neuronal synchronisation in more than 2 coupled brain areas. We will also analyse the influence of different interactions on the neuronal activities as proposed in the model of Potjans and Diesmann (2014).

## Data Availability Statement

The raw data supporting the conclusions of this article will be made available by the authors, without undue reservation.

## Author Contributions

All authors discussed the results and contributed to the final version of the manuscript.

## Conflict of Interest

The authors declare that the research was conducted in the absence of any commercial or financial relationships that could be construed as a potential conflict of interest.
